# Impact of low‐load resistance exercise with and without blood flow restriction on muscle strength, endurance, and oxidative capacity: A pilot study

**DOI:** 10.14814/phy2.16041

**Published:** 2024-06-18

**Authors:** Brett H. Davis, James E. Stampley, Joshua Granger, Matthew C. Scott, Timothy D. Allerton, Neil M. Johannsen, Guillaume Spielmann, Brian A. Irving

**Affiliations:** ^1^ School of Kinesiology Louisiana State University Baton Rouge Louisiana USA; ^2^ Pennington Biomedical Research Center Louisiana State University Baton Rouge Louisiana USA

**Keywords:** exercise, human, near‐infrared spectroscopy, NIRS, training

## Abstract

Low‐load resistance exercise (LLRE) to failure can increase muscle mass, strength, endurance, and mitochondrial oxidative capacity (OXPHOS). However, the impact of adding blood flow restriction to low‐load resistance exercise (LLBFR) when matched for volume on these outcomes is incompletely understood. This pilot study examined the impact of 6 weeks of single‐legged LLBFR and volume‐matched LLRE on thigh bone‐free lean mass, strength, endurance, and mitochondrial OXPHOS. Twenty (12 males and 8 females) untrained young adults (mean ± SD; 21 ± 2 years, 168 ± 11 cm, 68 ± 12 kg) completed 6 weeks of either single‐legged LLBFR or volume‐matched LLRE. Participants performed four sets of 30, 15, 15, and 15 repetitions at 25% 1‐RM of leg press and knee extension with or without BFR three times per week. LLBFR increased knee extension 1‐RM, knee extension endurance, and thigh bone‐free lean mass relative to control (all *p* < 0.05). LLRE increased leg press and knee extension 1‐RM relative to control (*p* = 0.012 and *p* = 0.054, respectively). LLRE also increased mitochondrial OXPHOS (*p* = 0.047 (nonparametric)). Our study showed that LLBFR increased muscle strength, muscle endurance, and thigh bone‐free lean mass in the absence of improvements in mitochondrial OXPHOS. LLRE improved muscle strength and mitochondrial OXPHOS in the absence of improvements in thigh bone‐free lean mass or muscle endurance.

## 1 INTRODUCTION

Blood flow‐restricted resistance exercise is an emerging training modality used in clinical settings (Ladlow et al., 2018; Loenneke et al., 2013; Patterson et al., 2017), athletics (Luebbers et al., 2014), and the general public (Clark et al., 2011; Yasuda et al., 2012). Low‐load (~20%–30% 1‐repetition maximum, 1 RM) blood flow‐restricted resistance exercise (LLBFR) has been shown to elicit gains in skeletal muscle mass, strength, and endurance (Loenneke et al., 2012; Yasuda et al., 2012). Clinically, LLBFR is an attractive rehabilitative approach to preserve muscle mass, strength, and endurance in individuals recovering from a musculoskeletal injury (Koc et al., 2022) and disuse atrophy (e.g., limb immobilization) (Cook et al., 2010). Moreover, LLBFR is a viable exercise therapy in older adults who may be unable to initiate a high‐load (~60%–80% 1‐RM) resistance exercise (HLRE) program at the onset of a training program (Cook et al., 2017). During LLBFR, a pneumatic cuff is placed on the proximal portion of the exercising limb and is inflated to prespecified limb occlusion pressure (60%–80% LOP) to limit arterial inflow and venous outflow, and then the muscle is exercised at low training loads. Moreover, LLBFR‐induced training adaptations in muscle mass, strength, and endurance are often comparable to the adaptations generally observed in response to HLRE (Loenneke et al., 2012). This alternative type of training could be highly beneficial for populations who would otherwise be contraindicated to perform HLRE, such as older adults or those with musculoskeletal impairments.

Blood flow‐restricted resistance exercise training studies have traditionally focused on muscle mass and strength adaptations (Shinohara et al., 1998; Yamanaka et al., 2012), with relatively few focusing on mitochondrial adaptations (Groennebaek et al., 2018; Petrick et al., 2019). Classically, endurance exercise has been the gold standard for inducing beneficial mitochondrial adaptations (Holloszy, 1967). In contrast, HLRE has been traditionally viewed as having minimal impact on mitochondrial adaptations (Groennebaek & Vissing, 2017). However, emerging data suggest that HLRE can also induce beneficial mitochondrial adaptations, particularly in sedentary adults, albeit to a lesser extent than endurance exercise (Porter et al., 2015). Recently, researchers have become increasingly interested in the potential impact of low‐load resistance exercise (LLRE) on mitochondrial adaptations. For example, a recent study showed that 10 weeks of LLRE effectively increased the protein content of some key mitochondrial proteins (e.g., DRP1 and SOD1) (Lim et al., 2019). Interestingly, increases in many of the mitochondrial proteins in response to LLRE were similar between the group that was volume matched with the HLRE and the group that completed the LLRE program to failure (Lim et al., 2019). Moreover, due to the enhanced metabolic stress induced by BFR, researchers have begun to examine whether BFR can enhance the beneficial effects of LLRE on mitochondrial remodeling and oxidative capacity (OXPHOS) (Groennebaek et al., 2018; Petrick et al., 2019). Mechanistically, exercise‐induced mitochondrial adaptations are mediated by acute homeostatic perturbations, which activate multiple cellular signal transduction pathways, leading to alterations in transcriptional regulation, protein synthesis, and protein breakdown (Egan & Sharples, 2023). Several LLRE metabolic perturbations are likely accentuated by BFR, including an accumulation of metabolic byproducts (e.g., lactate, Pi, Ca^++^, and H^+^), changes in the hormonal milieu, and reductions in cellular PO_2_ that likely activate molecular signal transduction pathways promoting mitochondrial adaptations (Ferguson et al., 2018; Lowery et al., 2014). Consistent with this notion, recent data suggest that 6 weeks of LLBFR performed to failure can improve mitochondrial OXPHOS in permeabilized muscle fibers measured using high‐resolution respirometry (Groennebaek et al., 2018). However, another study showed that 6 weeks of LLRE, but not LLBFR, when performed to failure, increased mitochondrial OXPHOS measured in permeabilized muscle fibers (Pignanelli et al., 2020). With just a few studies showing potentially conflicting effects of LLBFR on mitochondrial OXPHOS, particularly when compared to LLRE, further investigations are essential, as discussed in a recent viewpoint (Wernbom, 2024). A strength of the prior studies was the training sessions were performed to failure; however, as a result, the LLRE groups had significantly greater training volumes than the LLBFR. Exercise training‐induced changes in mitochondrial OXHPOS are not only sensitive to training intensity but also to training volumes. Therefore, in the present study, we aimed to examine the impact of 6 weeks of single‐legged LLRE and LLBFR matched for training volume on measures of muscle mass, strength, and mitochondrial OXPHOS. We hypothesized that both LLRE and LLBFR would result in improved muscle mass, strength, and mitochondrial OXPHOS in the trained compared to the untrained control leg and that the LLBFR would lead to greater gains in muscle mass, strength, and mitochondrial OXPHOS compared to LLRE.

## 2 METHODOLOGY

### 2.1 Participants

Twenty (12F/8M) untrained young adults (mean ± SD; 21 ± 2 years, 168 ± 11 cm, 68 ± 12 kg) completed this 6‐week exercise study. Participants had body mass indices (BMI) between 18.5 and 30 kg/m^2^ and had no medical conditions that would preclude them from physical activity based on the Physical Activity Readiness Questionnaire for Everyone (PARQ+).

### 2.2 Screening protocol

Potentially eligible participants completed an initial screening visit in the LSU Exercise Physiology Laboratory. The screening visit consisted of informed consent, self‐report medical history, the PARQ+, and the International Physical Activity Questionnaire. Participants then had their resting heart rate and blood pressure measured by an automated sphygmomanometer (Omron Intellisense Digital Blood Pressure Monitor). Finally, their height and body mass were measured to calculate their body mass index (BMI). Participants who reported engaging in resistance or aerobic activity >2 days/week within the past 3 months of screening were excluded. Participants were also excluded if they reported having diabetes, cardiovascular disease, blood clotting diseases, deep vein thrombosis, sickle cell trait or anemia, or any musculoskeletal or neurological disorders that would preclude them from exercise. Participants were excluded if they took prescription medications other than hormonal contraception. Finally, participants were excluded if they reported nicotine or excessive alcohol use (>14 drinks per week).

### 2.3 Study design

**FIGURE 1:**
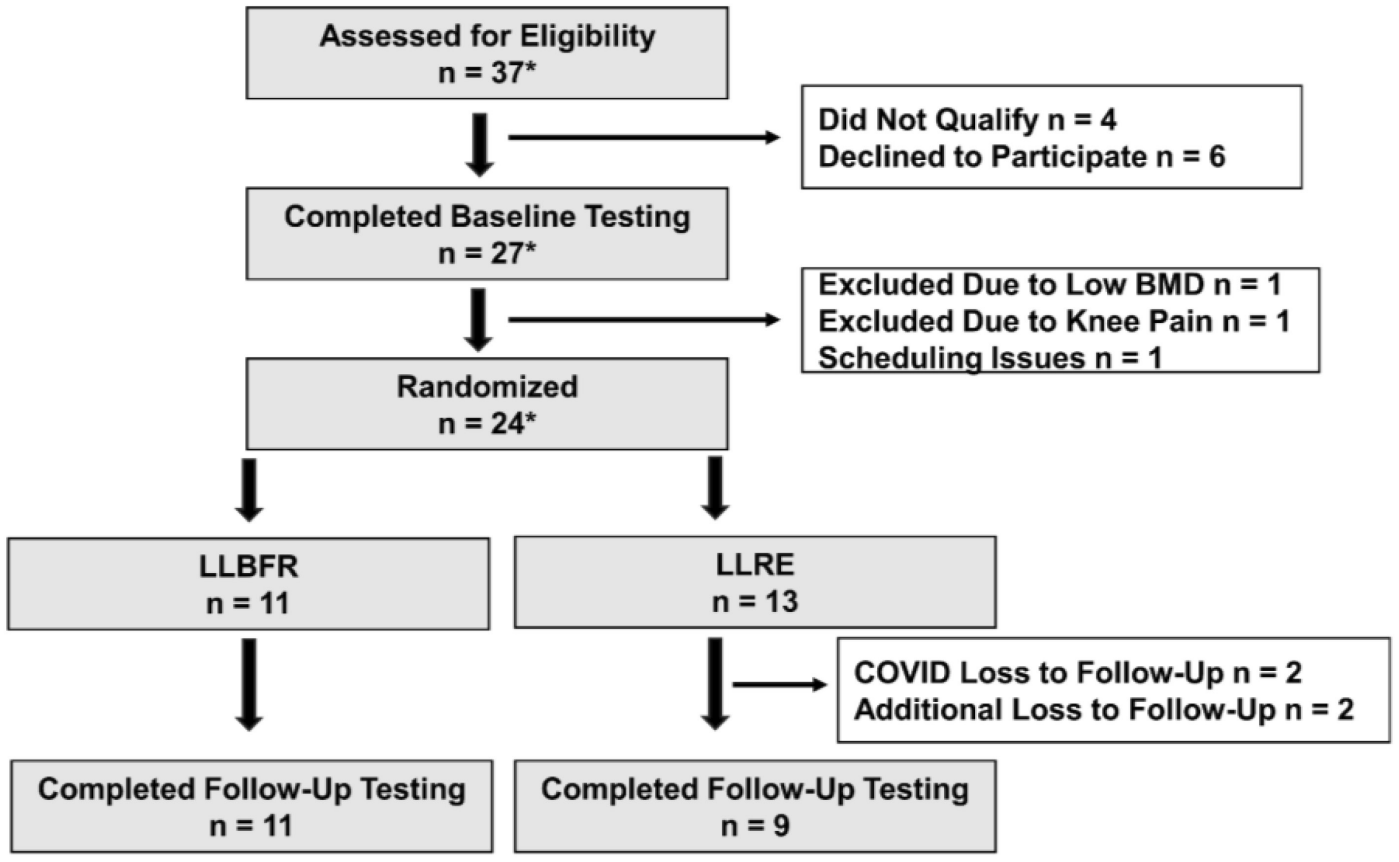
Consort diagram describing participant enrollment, randomization, and study completion. *Five of the participants were screened, baseline tested, and randomized; however, their participation was interrupted by COVID19. Three of those five participants returned after a 6–8 months washout and were reassessed for eligibility followed by new baseline testing and study participation. Initial randomization for those three participants was maintained.

The eligible participants were randomized into one of two 6‐week single‐legged exercise groups: (i) LLBFR or (ii) LLRE in a counterbalanced fashion. Moreover, the leg exercised was also randomized in a counterbalance fashion based on dominance, as the non‐exercised leg served as an internal control. Randomization schemes were prepared using Research Randomizer (https://www.randomizer.org). Figure 1 presents the CONSORT Diagram showing the overall distribution of the study. The participants were assessed before (pre‐training) and ~ 48–72 h after (post‐training) their last training session. Participants were asked to refrain from alcohol, caffeine, and exercise for at least 24 h before their assessment visits. Participants were asked to arrive at the LSU Exercise Physiology Laboratory after an overnight fast (≥10) between ~7:00 and 10:00 AM for their assessment visits. The details of the individual assessments are provided below.

### 2.4 Body composition assessment

Whole‐body dual‐energy X‐ray absorptiometry (DXA) scans were performed using a Hologic Horizon A DXA Scanner as previously described (Wong et al., 2023). The whole‐body scans were analyzed using Hologic Apex Software. The primary focus of the present DXA analyses was to assess changes in leg and thigh bone‐free lean mass between the trained and untrained control legs. The leg bone‐free lean mass in the trained and untrained legs was quantified using standard DXA segmentation protocols (Wong et al., 2023). The thigh bone‐free lean mass in the trained and untrained legs was quantified using the region of interest (ROI) analyses described previously (Hirsch et al., 2021).

### 2.5 Muscle strength and endurance assessments

Participants performed a 5‐minute warm‐up on a treadmill at 3 miles per hour before strength testing. Isometric knee extensor strength was measured (peak torque, N∙m) at a joint angle of 60° (0° = full knee extension). Isokinetic muscle strength was assessed at 60°/s using a Biodex System 3 (Shirley, NY). Briefly, the participants completed a short set of practice trials to familiarize themselves with the testing protocol, followed immediately by three maximal repetitions per test (1 min rest between repetitions). Finally, an isokinetic knee extensor endurance test comprised of 120 maximal voluntary contractions at 240°/s (one every 2 s for 4 min) was performed to measure the total work (i.e., a measure of absolute endurance) as previously described (Fitzgerald et al., 2020). In addition, we also determined the percent change in peak torque measured during the first 20 repetitions relative to the last 20 repetitions (i.e., relative endurance). The isokinetic strength and endurance tests were performed on both legs and conducted over a 70° range of motion (80–10°, 0° = full knee extension).

Skeletal muscle strength was also assessed using single‐legged 1‐RM tests for the leg extension and leg press. The participants were provided a dynamic warm‐up consisting of a load they could lift at least six times. The participants completed four to six trials of increasing weight, followed by 2–3 min of recovery, until they achieved their 1‐RM with proper form. Both legs were tested for leg extension and leg press 1‐RM, and the order of the tests remained the same for all participants and time points. Likewise, the same encouragement was given for each test. The 1‐RM tests were performed before the first and last exercise training session.

### 2.6 Skeletal muscle mitochondrial oxidative capacity assessment

We used continuous‐wave near‐infrared spectroscopy (Oxymon MKIII, Artinis Medical Systems, Netherlands) coupled with neuromuscular electrical stimulation (NMES) (Revolution Wireless, Chattanooga, TN) to measure skeletal muscle (*vastus lateralis*) oxidative capacity (NIRS‐OXPHOS) as previously described (Hanna et al., 2021; Ryan et al., 2012) with slight modifications in both the trained and untrained. The NIRS assessment was performed after the body composition assessment and before the muscle strength and endurance assessments. A detailed description of the methods is provided in the Supplementary Methods S1. In brief, the muscle oxygen consumption *mV̇O*_*2*_ recovery kinetics following the NMES were fit using custom‐built MATLAB (The Mathworks, Natick, MA) routines written by Terence Ryan (Ryan et al., 2012; Ryan, Brizendine, & McCully, 2013). The *mVO*_*2*_ for each recovery occlusion was measured after applying a blood volume correction and an ischemic calibration as described previously (Ryan et al., 2012; Ryan, Brizendine, & McCully, 2013). The *mVO*_*2*_ slopes were subsequently fit to the following mono‐exponential function: *y* = End – (Δ × e^−kt^) within the custom‐built MATLAB program (Ryan et al., 2012; Ryan, Brizendine, & McCully, 2013). In brief, the “*y*” represents (blood volume corrected) *mVO*_*2*_, Δ represents the change in *mVO*_*2*_ from rest to the end muscle activation (e.g., NMES), and *k* represents the rate constant, while “*t*” is the time (Ryan et al., 2012; Ryan, Brizendine, & McCully, 2013). The primary outcome from the present analysis was the rate constant from the *mVO*_*2*_ recovery kinetics—the rate constant equals 1/t*60 sec or 1/min, which reflects mitochondrial OXPHOS capacity. Prior studies by Ryan et al. have validated the NIRS OXPHOS rate constants against both in vivo (e.g., NMR spectroscopy) and in situ (e.g., permeabilized muscle fibers) (Ryan et al., 2014; Ryan, Southern, et al., 2013). The rate constants included in the present analyses are the average of duplicate/triplicate measures. Rate constants were excluded from the present analyses if the *R* ^2^ of the monoexponential fit was <0.9, the coefficient of variation exceeded 20%, or the mVO_2_ recovery kinetics failed to fit a monoexponential curve. After excluding low‐quality fits, the average *R* ^2^ was ~0.98, and the within‐day test–retest coefficient of variation was ~9%. Although not directly measured in the present study, the between‐day test–retest coefficient of variation has been reported to be between 10% and 14% using a similar NIRS OXPHOS protocol (Hanna et al., 2021). As a result, 8 participants per treatment group had complete pre‐ and post‐intervention NIRS OXPHOS data.

### 2.7 Exercise intervention

#### 2.7.1 LLBFR

Participants were fitted with the Easi‐Fit Tourniquet Cuff designed for the Delfi Personalized Tourniquet System (PTS) for BFR training (Delfi Medical Innovations) on the proximal part of the thigh. The cuff was inflated to 60% of LOP using the PTS. Participants then performed four sets of 30, 15, 15, and 15 repetitions at 25% of their 1‐RM for the single‐legged leg press. The LOPs, sets, and repetitions were based on the BFR consensus guidelines (Patterson et al., 2019). Participants were given an inter‐set rest interval of 60 s. After the single‐legged leg press, the cuff was deflated, and participants were given a 3‐min rest interval. The cuff was then reinflated to 60% LOP at the start of the knee extension exercise. Participants then performed four sets of 30, 15, 15, and 15 repetitions at 25% of their 1‐RM for the single‐legged knee extension. Participants were given an inter‐set rest interval of 60 s. After the single‐legged knee extension, the cuff was deflated. The length of exposure to BFR was about 6 min per exercise and 12 min per training session. The Delfi PTS uses Doppler to measure and precisely regulate the LOP before and during exercise.

#### 2.7.2 LLRE

Participants completed the same protocol as LLBFR without the blood flow restriction, consisting of single‐legged leg press and knee extension exercises consisting of four sets of 30, 15, 15, and 15 repetitions at 25% of their 1‐RM for each exercise with a 60‐s rest intervals between sets, and a 3‐min rest interval between exercises.

For both interventions, participants completed only two sets of 30 and 15 repetitions of each exercise during Week 1 and three sets of 30, 15, and 15 repetitions of each exercise during Week 2 to provide an acclimation period. During Weeks 3–6, participants performed four sets of 30, 15, 15, and 15 repetitions as described above. The relative load was held constant throughout the 6‐week intervention (e.g., 25% of pretraining 1‐RM). Therefore, the increase in training volume during the study was due to adding additional sets during Weeks 1–3. At the end of each set, ratings of perceived exertion were collected using the Borg [6‐20] (Borg, 1982). Overall, the target number of sessions over the 6‐week training intervention was 18 sessions. However, a few participants had to add a few training sessions to accommodate their final testing.

### 2.8 Statistical analysis

Data were analyzed using JMPro 17 (SAS Inc., Cary, NC, USA) and are presented as mean ± SD or LSMEANS ±95% confidence intervals where appropriate. Mixed‐effects analysis of variance models were used to assess the impact of the main effect of training (trained vs. untrained control leg), group (LLRE vs. LLBFR), and their interactions on the pre‐ to post‐training changes (Δ) in study outcomes. A random effect ID nested in group (ID[group]) was included in each model to account for repeated measures. We also report the *a priori* linear contrasts of interest from the mixed‐effects models (ΔTrained – ΔUntrained Control) within each group, as well as nonparametric equivalents to serve as a sensitivity analysis. For the primary outcomes, the effect sizes were estimated using the test statistics (*F*‐values, degrees of freedom, and accounting for pairing) for our a priori linear contrasts of trained – untrained control comparisons (ΔTrained – ΔUntrained Control) using the “f_to_d” function in the *effectsize* package (Ben‐Shachar et al., 2020) within R. As a pilot study, this study was initially designed to estimate effect sizes for the primary outcomes using an *n* = 12 per group. However, due in part to the COVID19 pandemic, we stopped the present study with 20 completed participants (*n* = 9 for LLRE and *n* = 11 for LLBFR). One advantage of the single‐legged (or unilateral) exercise model is enhanced statistical power compared to the parallel study design (MacInnis et al., 2017). With the single‐leg study design, an effect size of 1.0 can be detected with ~80% power at an alpha of 0.05 with 10–12 participants per group (MacInnis et al., 2017).

## 3 RESULTS

### 3.1 Baseline physical activity and body composition

At baseline (pretraining), the participants accumulated 287 ± 574, 160 ± 285, and 2622 ± 3043 MET‐min/week (*n* = 18) for moderate, vigorous, or total physical activity based on the IPAQ questionnaire, with no significant differences between groups (all *p* > 0.05). Two participants had IPAQ questionnaires filled out incorrectly, and these questionnaires were excluded. At baseline, the participants had a body fat percent of 25.7% ± 8.1%, fat mass of 17.4 ± 5.8 kg, and lean mass of 51.2 ± 11.2 kg, with no significant differences between intervention groups (all *p* > 0.05).

### 3.2 Baseline strength, endurance, leg and thigh bone free lean mass, and OXPHOS

Table 1 presents the pretraining skeletal muscle strength, knee extensor endurance, leg bone‐free lean mass, thigh bone‐free lean mass, and mitochondrial OXPHOS before training stratified by treatment group and training leg. No significant differences were noted between the trained and untrained control legs at baseline (all *p* > 0.05), except for the knee extension 1‐RM that was lower in the trained compared to the untrained leg within the LLRE group at baseline (*p* = 0.025).

**TABLE 1:** Baseline (pretraining) values for muscle strength, endurance, leg bone‐free lean mass, thigh bone‐free lean mass, and mitochondrial oxidative capacity (OXPHOS) stratified by the group and training leg.

Outcome	LLRE (*n* = 9)		LLBFR (*n* = 11)	
	Untrained	Trained	Untrained	Trained
Leg press 1‐RM, kg	112 ± 31	108 ± 31	85 ± 29^b^	92 ± 38
Knee extension 1‐RM, kg	62 ± 18	58 ± 18*	45 ± 17^b^	48 ± 20
Isometric torque, N∙m	185 ± 55	185 ± 42	163 ± 41	165 ± 45
Isokinetic torque, N∙m	147 ± 33	150 ± 39	143 ± 40	139 ± 37
Total work^c^, J	6,755 ± 2,096^a^	6,176 ± 1,884^a^	5,926 ± 2,196	5,776 ± 2,742
Change in peak torque^c^, %	−35.0 ± 8.4^a^	−35.0 ± 11.2^a^	−30.5 ± 18.3	−27.7 ± 21.3
Leg bone‐free lean mass, kg	8.8 ± 1.9	8.7 ± 2.0	7.9 ± 2.0	7.8 ± 2.0
Thigh bone‐free lean mass, kg	6.2 ± 1.3	6.2 ± 1.4	5.5 ± 1.3	5.4 ± 1.2
NIRS OXPHOS rate constant, min^−1^	1.3 ± 0.5^a^	1.2 ± 0.3 ^a^	1.4 ± 0.7 ^a^	1.4 ± 0.3 ^a^

*Note*: Data are mean ± SD.

^a^ *n* = 8.

^b^ *n* = 9.

^c^ Measured during the 4‐min knee extensor endurance test.

* *p* < 0.05.

### 3.3 Session attendance, training volumes, and RPEs

The LLRE group completed 17 ± 1 training sessions, while the LLBFR group completed 18 ± 1 training sessions (*p* = 0.005). The overall training volume achieved by the participants in the LLRE group was 48,459 ± 14,684 kg, while the LLBFR group completed 43,259 ± 16,948 kg (*p* = 0.478). Table 2 presents the exercise training data stratified by group (LLRE vs. LLBFR) and individual exercises (knee extension vs. leg press).

**TABLE 2:** Exercise data presented by training group (*n* = 11 LLBFR, *n* = 9 LLRE).

Variable	Group	Mean ± SD	*p*‐value*
Knee extension volume per session (kg)	LLRE	876 ± 423	
	LLBFR	795 ± 347	0.644
Leg press volume per session (kg)	LLRE	1,828 ± 537	
	LLBFR	1,562 ± 651	0.340
Knee extension total volume (kg)	LLRE	17,082 ± 5,782	
	LLBFR	14,617 ± 6,191	0.374
Leg press total volume (kg)	LLRE	31,396 ± 9,974	
	LLBFR	28,642 ± 11,483	0.579
Knee extension RPE (6–20)	LLRE	11.8 ± 1.8	
	LLBFR	12.7 ± 1.0	0.162
Leg press RPE (6–20)	LLRE	10.0 ± 1.6	
	LLBFR	11.4 ± 1.5	0.071

* *t*‐test assuming equal variances.

### 3.4 Exercise training‐induced changes in leg and thigh bone‐free lean mass

**FIGURE 2:**
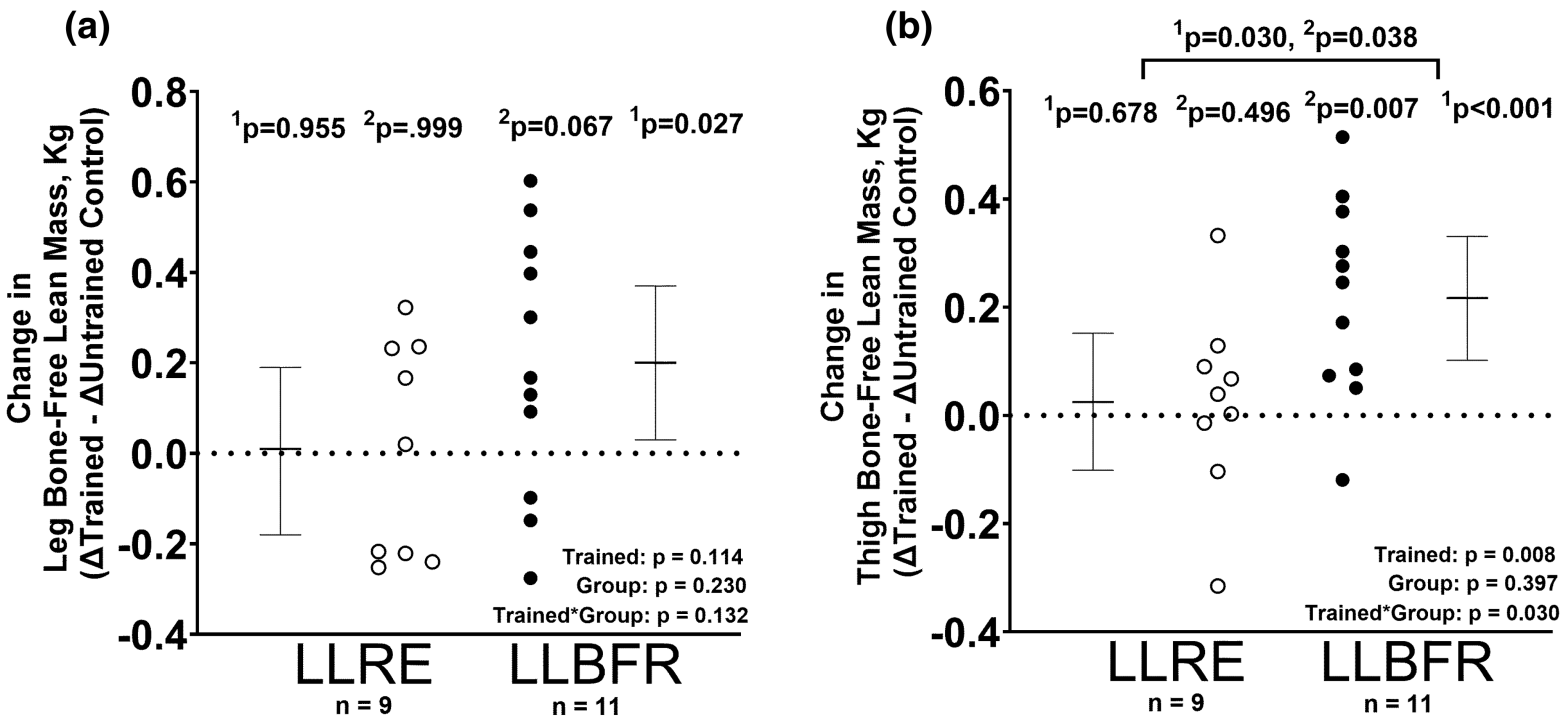
Pre‐ to post‐training changes (Δ) within the trained relative to the untrained control leg (ΔTrained –Untrained Control) are presented for leg bone‐free lean mass (a) and thigh bone‐free lean mass (b) measured using DXA. The trained leg completed 6 weeks of either low‐load resistance exercise (LLRE) or low‐load blood flow‐restricted resistance exercise (LLBFR) 3 days per week. Data are LSMEANS±95% confidence intervals using linear mixed‐effects models to test for the main effects of training (trained vs. untrained), group (LLRE vs. LLBFR), and their interaction. Individual data points are also provided (open circles = LLRE, closed circles = LLBFR). ^1^ *p*‐Values are based on linear contrasts from the mixed models, while the ^2^ *p*‐values are based on nonparametric post hoc tests.

LLBFR increased the leg and thigh bone‐free lean mass in the trained relative to the untrained control leg [effect size: 0.57 (0.06, 1.06), *p* = 0.027 and effect size: 0.94 (0.38, 1.48), *p* = 0.001, respectively] (Figure 2a,b). In contrast, LLRE did not increase either leg or thigh bone‐free lean mass in the trained relative to the untrained control leg [effect size: 0.01 (−0.45, 0.48), *p* = 0.955 and effect size: 0.10 (−0.37, 0.56), *p* = 0.678, respectively] (Figure 2a,b). Of note, the training‐induced increases in thigh bone‐free lean mass in the LLBFR were significantly greater than the change observed in the LLRE group (*p* = 0.030). Supplemental Table S1 presents the pre‐ to post‐training changes stratified by leg (e.g., LLRE trained leg, LLRE untrained leg, LLBFR trained leg, and LLBFR untrained leg).

### 3.5 Exercise training‐induced changes in muscle strength and endurance

**FIGURE 3:**
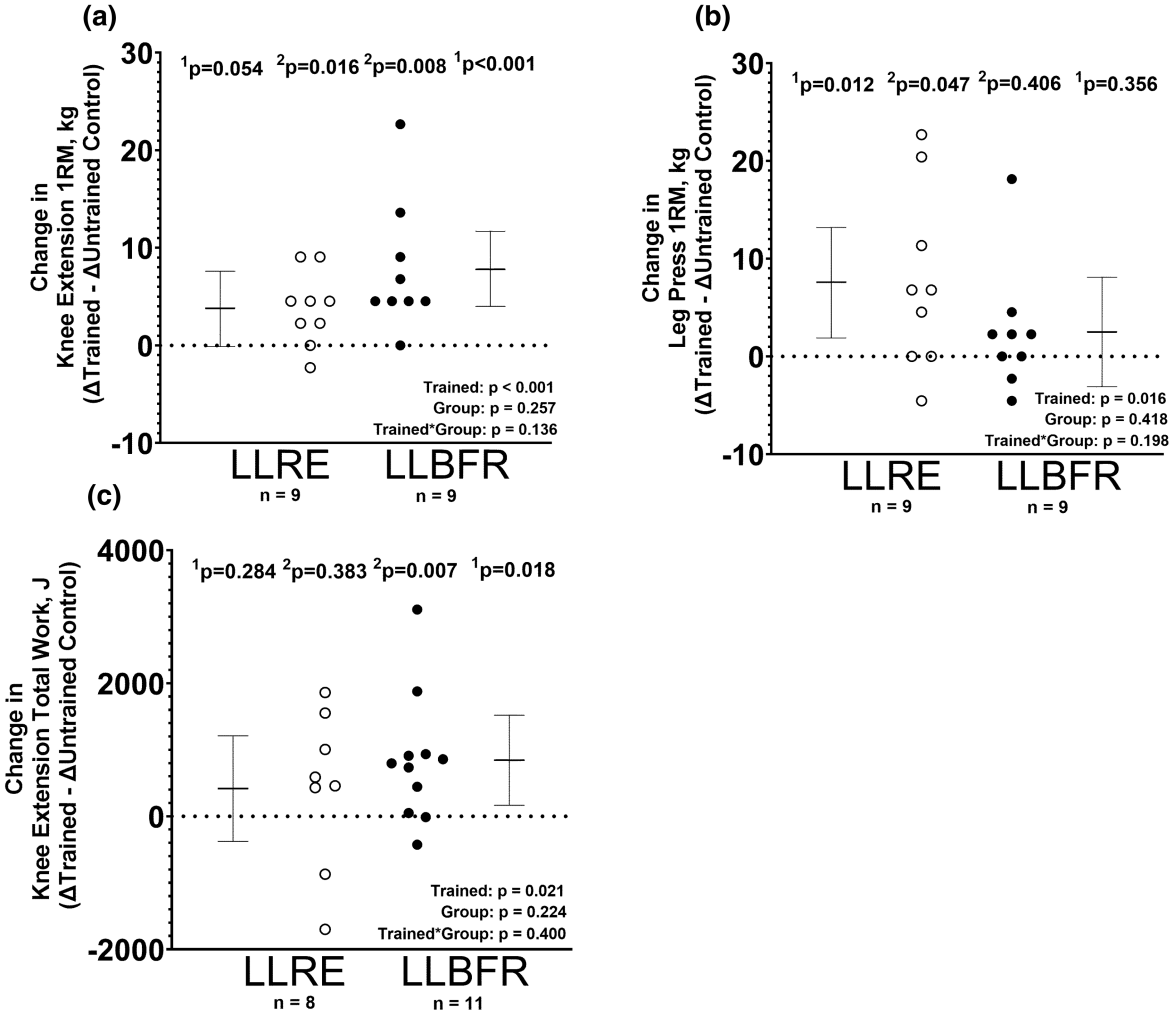
Pre‐ to post‐training changes (Δ) in the trained relative to the untrained control leg (Δ Trained –Δ Untrained Control) are presented for knee extension 1‐RM (a), leg press 1‐RM (b), and total work measured during a 4‐min knee extensor endurance test (c). Data are LSMEANS ± 95% confidence intervals using linear mixed‐effects models to test for the main effects of training (trained vs. untrained), group (LLRE vs. LLBFR), and their interaction. Individual data points are also provided (open circles = LLRE, closed circles = LLBFR). ^1^ *p*‐values are based on linear contrasts from the mixed models, while the ^2^ *p*‐values are based on nonparametric post hoc tests.

Neither LLRE nor LLBFR changed the isometric knee extensor strength in the trained relative to the untrained control leg [effect sizes: 0.003 (−0.46, 46) and 0.07 (−0.39, 0.53), respectively, all *p* > 0.05]. Likewise, neither LLRE nor LLBFR significantly changed the isokinetic strength measures in the trained relative to the untrained control leg [effect sizes: 0.08 (−0.38, 0.54) and 0.02 (−0.44, 0.48), respectively, all *p* > 0.05]. LLBFR increased the knee extension 1‐RM in the trained relative to the untrained control leg [effect size: 1.08 (0.45, 1.68), *p* < 0.001] (Figure 3a). However, the increase in the knee extension 1‐RM in the trained compared to the untrained control leg in the LLRE group was not significant [effect size: 0.52 (−0.01, 1.04), *p* = 0.054] (Figure 3a). As expected, LLRE increased the leg press 1‐RM in the trained relative to the untrained control leg [effect size: 0.71 (0.16, 1.25), *p* = 0.012] (Figure 3b). However, LLBFR did not increase the leg press 1‐RM in the trained relative to the untrained control leg [effect size: 0.24 (−0.26, 0.73), *p* = 0.356] (Figure 3b). LLBFR increased the total work performed during the 4‐min knee extensor endurance test in the trained relative to the control leg [effect size: 0.64 (0.11, 1.15), *p* = 0.018] (Figure 3c). LLRE did not significantly increase the total work during the 4‐min knee extensor endurance test in the trained relative to the control leg [effect size: 0.27 (−0.22, 0.75), *p* = 0.284] (Figure 3c). Training did not significantly affect the percent change in peak torque during the 4‐min endurance test (*p* > 0.05).

### 3.6 Exercise training‐induced changes in mitochondrial oxidative capacity (OXPHOS)

**FIGURE 4:**
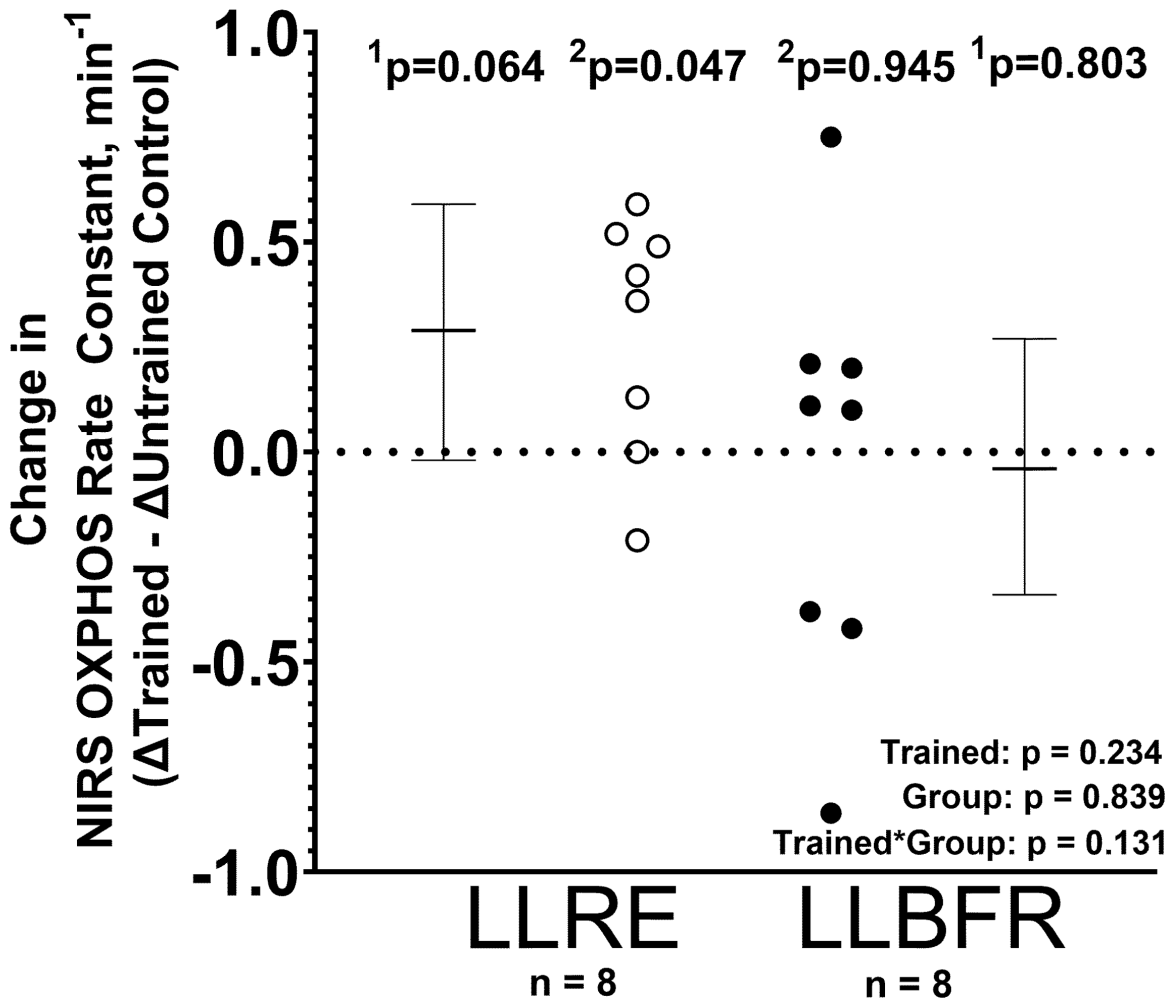
Pre‐ to post‐training changes (Δ) in the trained relative to the untrained control leg (ΔTrained –ΔUntrained Control) are presented for the skeletal muscle mitochondrial oxidative capacity (OXHPHOS) rate constant measured using near‐infrared spectroscopy (NIRS). The trained leg completed 6 weeks of either low‐load resistance exercise (LLRE) or low‐load blood flow‐restricted resistance exercise (LLBFR) 3 days per week. Data are LSMEANS ± 95% confidence intervals using linear mixed‐effects models to test for the main effects of training (trained vs. untrained), group (LLRE vs. LLBFR), and their interaction. Individual data points are also provided (open circles = LLRE, closed circles = LLBFR). ^1^ *p*‐values are based on linear contrasts from the mixed models, while the ^2^ *p*‐values are based on nonparametric post hoc tests.

Neither LLRE nor LLBFR increased the skeletal muscle NIRS‐OXPHOS rate constant in the trained relative to the untrained control leg [effect size: 0.54 (−0.03, 1.09), *p* = 0.064 and effect size: −0.07 (−0.46, 0.59), *p* = 0.803, respectively] (Figure 4).

## 4 DISCUSSION

The present pilot study examined the effects of 6 weeks of LLRE with and without BFR matched for training volume on leg and thigh bone‐free lean mass, strength, endurance, and mitochondrial oxidative capacity using a single‐legged (within‐subject) training model. Our main findings were that LLBFR increased knee extension 1‐RM, knee extensor total work measured during a 4‐min knee extensor endurance test, leg bone‐free lean mass, and thigh bone‐free lean mass in the trained compared to the untrained control leg. Contrary to our hypothesis, we did not observe an increase in mitochondrial OXPHOS measured using NIRS in response to LLBFR in the trained relative to the untrained control leg. LLRE increased the leg press 1‐RM, along with a nonsignificant increase in knee extension 1‐RM, total work during the 4‐min knee extensor endurance test, and mitochondrial OXPHOS measured by NIRS in the trained relative to the untrained control leg. However, non‐fatiguing LLRE did not significantly change the leg or thigh bone‐free lean mass in the trained relative to the untrained control leg. These findings are consistent with the literature showing that LLBFR effectively increases muscle mass, strength, and endurance (Loenneke et al., 2012; Martin‐Hernandez et al., 2013).

Although LLBFR has been shown to increase muscular strength and hypertrophy, there are relatively few studies on short‐term mitochondrial adaptations. Due to the increased metabolic stress imposed by BFR, LLBFR has been hypothesized to enhance the mitochondrial adaptations observed in response to LLRE (Ferguson et al., 2018; Groennebaek et al., 2018; Pignanelli et al., 2020; Saatmann et al., 2021). Emerging data suggests that LLBFR performed to failure increases mitochondrial OXPHOS in some (Groennebaek et al., 2018), but not all studies. (Pignanelli et al., 2020) For example, recent data from Groennebaek et al. (2018) suggested that 6 weeks of LLBFR, when performed to failure, increased mitochondrial OXPHOS in permeabilized muscle fibers. In addition to showing improvements in mitochondrial OXPHOS with LLBFR, Groennebaek et al. (2018) elegantly showed LLBFR‐induced increases in mitochondrial protein synthesis using deuterium labeling. In contrast, recent data from Pignanelli et al. (2020) demonstrated that 6 weeks of LLRE, but not LLBFR, increased mitochondrial OXPHOS measured in permeabilized muscle fibers when performed to failure. One potential difference between these two studies is the LOP, which was higher in the later study (e.g., 60% vs. 50%). As a result, the higher LOP could have blunted the exercise‐induced production of mitochondrial H_2_O_2_, a key mediator of mitochondrial adaptations (Petrick et al., 2019). Consistent with observations of Pignanelli et al. (2020), the present results suggest that 6 weeks of (non‐fatiguing) LLRE increased mitochondrial OXPHOS (i.e., NIRS OXPHOS rate constant) in the trained relative to the untrained control leg. Moreover, our results also did not show an exercise training‐induced increase in mitochondrial OXPHOS following 6 weeks of LLBFR in the trained relative to the untrained control leg. Like Pignanelli et al. (2020), the present study used an LOP of 60%, which also could have blunted exercise‐induced production of H_2_O_2_. The prior studies showing improvements in mitochondrial OXPHOS capacity following either short‐term LLRE or LLBFR used permeabilized muscle fibers (Groennebaek et al., 2018; Pignanelli et al., 2020; Porter et al., 2015), which are measured independent of potential changes in skeletal microvascular perfusion. In contrast, the in vivo NIRS OXPHOS measurements used in the present study are partially dependent on microvascular perfusion, which may be altered by BFR training. Future in vivo studies are needed to examine both changes in microvascular perfusion and mitochondrial OXPHOS capacity.

Our study showed increases in leg and thigh bone‐free lean mass in the trained compared to the untrained control leg in response to LLBFR, while no training‐induced increases were observed in response to LLRE. Moreover, the increase in thigh bone‐free lean mass was also significantly greater in the LLBFR group compared to the LLRE group. The present results support the hypothesis that skeletal muscle hypertrophy likely occurs earlier in the neuromuscular adaptive response to LLBFR, particularly when compared to volume‐matched LLRE (Loenneke et al., 2012). Similarly, a recent study in older adults also showed an ~1.5% increase in thigh bone‐free lean mass following 6 weeks of LLBFR relative to control (*p* < 0.07) when performed to failure (Wang et al., 2023). We recognize that bone‐free lean mass is only a proxy for skeletal muscle mass, which could be affected by exercise due to transient cellular swelling and chronic cellular hypertrophy. However, as the post‐training DXA scans were acquired ~48–72 h after the last training bout, the likelihood of transient cellular swelling impacting our measurements is unlikely. Mechanistically, transient BFR‐induced myocellular swelling has been postulated to contribute to chronic hypertrophy, as shown by increased CSA following BFR interventions (Lowery et al., 2014; Wernbom et al., 2013). Recent histological data suggest that 6 weeks of LLBFR and HLRE lead to fiber level muscle hypertrophy in healthy young adults when performed to failure (Libardi et al., 2024). A meta‐analysis reviewing LLBFR showed that adding BFR to LLRE significantly increased hypertrophy comparable to HLRE with no difference between the two training conditions (effect sizes LLBFR = 0.39 vs. HLRE = 0.36 *p* > 0.05) (Loenneke et al., 2012). Also, 6 weeks of LLBFR has been shown to induce similar gains in similar gains in muscle hypertrophy compared to LLRE when both are performed to failure despite lower total training volumes (Farup et al., 2015). In contrast, the present study indicates that when LLRE is matched to LLBFR for total volume, only LLBFR results in significant increases in leg and thigh bone‐free lean mass in response. Thus, with respect to muscle hypertrophy, non‐fatiguing LLRE appears insufficient to stimulate muscle hypertrophy.

Since the first publication of the impact of LLBFR on muscle strength in 1998 (Shinohara et al., 1998), many studies have examined the efficacy of BFR on muscle strength outcomes. In the present study, LLBFR increased muscle strength, particularly knee extension 1‐RM, in the trained leg relative to the untrained control leg. With LLBFR, strength measurements have consistently increased, but usually not as high as traditional HLRE (Loenneke et al., 2012). In addition, LLBFR also improved the total work performed during the 4‐min knee extension endurance test in the trained compared to the untrained control leg, which is indicative of improvements in absolute endurance. However, there was no change in the relative endurance (e.g., % change in peak torque) during the 4‐min knee extensor endurance test following LLBFR training. A recent study reported that 6 weeks of LLBFR performed to failure increased the number of knee extension repetitions at 30% of 1‐RM that could be completed to failure among older adults (Wang et al., 2023). Of note, the post‐training measurements were conducted using 30% of the post‐training 1‐RM (Wang et al., 2023); as such, the results likely reflect improvements in both absolute and relative muscular endurance. Similar to the present study, Pignanelli et al. (2020) also showed improvements in knee extensor endurance following 6 weeks of LLBFR to failure despite nonsignificant changes in mitochondrial OXPHOS. In another recent study (Ida & Sasaki, 2024), which performed 6 weeks of low‐load (30% 1RM) elbow flexion training with (~40% LOP) and out BFR improved elbow flexor endurance, but not to the same degree as LLRE alone when performed to 1‐set to failure. Of note, the participants also only trained 2 days per week (Ida & Sasaki, 2024). Moreover, the results of this study showed that exercise volume was a key factor in improving local muscle endurance, particularly when training volumes are already relatively small (Ida & Sasaki, 2024). Our results suggest that when exercise volume is matched, LLBFR increases muscle endurance as well, if not better than LLRE.

## 5 STRENGTHS

One of the strengths of the present study was the use of a single‐legged (unilateral) exercise model. The single‐legged model increases statistical power and requires fewer participants than a more traditional parallel group design and half the time as using a traditional crossover design (MacInnis et al., 2017). Another strength of this study was our compliance for those who completed the intervention and post‐intervention data assessments. The average session attendance was ~95% for LLRE and ~100% for LLBFR.

## 6 LIMITATIONS

One limitation of the present study was that it started before the COVID19 pandemic and was completed after the pandemic. As a result, a few participants were lost to follow‐up due to the university‐related shutdowns. Moreover, some participants may have been exposed to COVID19 before or during our study without their knowledge. COVID19 has been reported to cause impairments in mitochondrial and microvascular function (Colosio et al., 2023; Trinity et al., 2021). Although NIRS is an accepted method of measuring mitochondrial OXPHOS, NIRS does have a few limitations. For example, in a few of our participants, the NIRS tests did not pass quality control checks, so only a subset of the participants (8 per group) were available for final analysis. Moreover, NMES also tends to activate more fast‐twitch fibers than the more traditional voluntary exercise (Gregory & Bickel, 2005), consistent with our lower pretraining rate constants. Finally, concerning NIRS, Pilotto et al. (2022) recently published a more technically advanced NIRS protocol that can measure both OXPHOS and oxygen diffusion capacity, which could have enhanced our current study. Also, our relative loads were not adjusted for possible strength increases throughout the 6 weeks. However, we increased the training volume over the first few weeks by adding additional sets. The initial training acclimation period meant the effective training volume period was reduced by 33%. The 6‐week intervention may not have been long enough to see adaptations in all outcome measures. The low training volumes could have significantly impacted the changes in the leg press strength. In addition, we also recognize that the results of the present study may not directly translate LLRE performed to fatigue. Finally, the present study did not have a HLRE group to compare our findings.

## 7 CONCLUSIONS

Our study showed that 6 weeks of LLBFR was sufficient to increase knee extension 1‐RM, knee extensor total work measured during a 4‐min knee extensor endurance test, leg bone‐free lean mass, and thigh bone‐free lean mass. Although we did not observe changes in mitochondrial function as measured by NIRS in the LLBFR, we did see an increase within the LLRE group, which is consistent with the current literature. LLRE also improved muscle strength in the absence of improvements in thigh bone‐free lean mass or muscle endurance. Future studies should examine the impact of LLBFR on OXPHOS and oxygen diffusion capacity in more sedentary and older populations.

## AUTHOR CONTRIBUTIONS

Brett H. Davis, Timothy D. Allerton, Neil M. Johannsen, Guillaume Spielmann, and Brian A. Irving were involved in study design and methodology; Brett H. Davis, James E. Stampley, Joshua Granger, Matthew C. Scott, Neil M. Johannsen, Guillaume Spielmann, and Brian A. Irving were involved in data collection and analysis; Brett H. Davis was involved in writing—original draft preparation; all authors were involved in writing—review and editing. All authors have read and agreed to the published version of the manuscript.

## CONFLICT OF INTEREST STATEMENT

As noted above, Delfi, Inc. provided their Personalized Tourniquet System for use in the present study. However, Delfi, Inc. had no input in this manuscript's data analysis, interpretation, or writing of the present manuscript.

## ETHICS STATEMENT

The study protocol and consent form were approved by the Louisiana State University (LSU) Institutional Review Board (LSU IRB3934). The study was registered at clinicaltrials.gov (NCT03723226). All participants provided written and informed consent before any assessments were conducted.

## Supporting information


Table S1.

Data S1:

## Data Availability

Data are available upon request.
