# Supplementary material for: Impact of low‐load resistance exercise with and without blood flow restriction on muscle strength, endurance, and oxidative capacity: A pilot study
Source: Physiol Rep. 2024 Jun 18;12(12):e16041. doi: 10.14814/phy2.16041 (PMC11184470; doi:10.14814/phy2.16041)
Supplement: Supplementary file 1 — Table S1. [file PHY2-12-e16041-s002.docx]

| Supplemental Table 1. The pre- to post-training change scores (Δ) for muscle strength, endurance, leg bone-free lean mass, thigh bone-free leant mass, and mitochondrial oxidative (OXPHOS) capacity stratified by group and training leg. | | | | |
| --- | --- | --- | --- | --- |
|  | LLRE (n=9) | | LLBFR (n=11) | |
| Outcome | Control | Trained | Control | Trained |
| Δ Leg Press 1-RM, kg | -3.5±13.7 | 4.0±11.8 | 4.3±14.1 ^b^ | 6.8±16.3^b^ |
| Δ Knee Extension 1-RM, kg | 1.5±6.1 | 5.3±3.9 | 2.0±5.1^b^ | 9.8±5.8^b^ |
| Δ Isometric Torque, N∙m | 11.3±16.2 | 11.4±38.0 | -0.4±23.7 | 2.8±27.0 |
| Δ Isokinetic Torque, N∙m | 10.3±19.9 | 13.8±33.4 | -8.0±14.7 | -7.5±15.1 |
| Δ Total Work^c^, J | -377.0±658.7^a^ | 39.4±1074.0^a^ | -193.6±584.3 | 649.3±1033.8 |
| Δ Change in Peak Torque^c^, % | -7.7±13.3^a^ | -6.9±22.4^a^ | -0.7±11.2 | 2.8±22.5 |
| Δ Leg Bone-Free Lean Mass, kg | 0.02±0.23 | 0.02±0.32 | 0.08±0.40 | 0.27±0.24 |
| Δ Thigh Bone-Free Lean Mass, kg | 0.00±0.22 | 0.02±0.27 | -0.01±0.21 | 0.21±0.28 |
| Δ NIRS OXPHOS Rate Constant, min^-1^ | -0.11±37^a^ | 0.18±0.34 ^a^ | 0.03±0.22 ^a^ | -0.01±0.39 ^a^ |
| Data are mean±SD; a: n =8, b: n = 9, c: measured during the 4-min knee extensor endurance test. | | | | |
