# Supplementary material for: Impact of low‐load resistance exercise with and without blood flow restriction on muscle strength, endurance, and oxidative capacity: A pilot study
Source: Physiol Rep. 2024 Jun 18;12(12):e16041. doi: 10.14814/phy2.16041 (PMC11184470; doi:10.14814/phy2.16041)
Supplement: Supplementary file 2 — Data S1: [file PHY2-12-e16041-s001.docx]

Supplementary Methods

Skeletal Muscle Oxidative Capacity Assessment. An Easi-Fit Tourniquet Cuff (Delfi Medical Innovations) specifically adapted to work with the Hokanson Rapid Cuff Inflation System (Hokanson E20, Bellevue WA) was placed proximal to the leg being measured. Participants laid supine on an adjustable therapy table (Athletic Edge) with their knee slightly flexed. B-Mode ultrasound (LOGIQe, GE Healthcare, Little Chalfont, UK) was performed to assess adipose tissue thickness and muscle thickness of the *vastus lateralis* at the site of the NIRS optode placement. The continuous-wave NIRS optodes (Oxymon MKIII, Artinis Medical Systems) were subsequently placed perpendicular to the *vastus lateralis* just anterior to the iliotibial band and ~1/3 the distance between the top of the patella and the greater trochanter of the test leg. The inter-optode distance of the shallow optodes was set at ~2 times the mid-muscle depth determined by B-mode ultrasound (~40 mm, range of 35-45 mm). The optodes were secured in place using double-sided medical tape, single-sided medical tape, and an elastic Velcro strap. A Neuromuscular Electric Stimulation (NMES) (Chattanooga, Revolution Wireless) pad was placed flush with the vastus lateralis just distal to the tourniquet and proximal to the NIRS optodes. A second NMES pad was placed just distal the NIRS optodes.

During the NIRS-OXPHOS test, the Oxymon MKIII (Artinis Medical Systems, Netherlands) continuous-wave NIRS system equipped with laser diodes emitting at ~761 nm and ~847 nm was used to monitor real-time changes in oxygenated (O_2_Hb), deoxygenated (HHb), and total hemoglobin (tHb) at 10 Hz. The rapid cuff inflation system (Hokanson E20, Bellevue, WA) was controlled using a custom-built controller and Labview Software algorithms. Resting muscle oxygen consumption (mVO_2_) was measured in triplicate using arterial occlusions by inflating the blood pressure cuff to (~300 mmHg) for 30 seconds with at least 120 seconds between each measurement. An ischemic calibration procedure followed to calculate mVO_2_ corrected for blood volume as previously described ^1, 2^, which includes a 30-sec NMES of the *vastus lateralis* immediately followed by 5-minute arterial occlusion (~300 mmHg) and subsequent reactive hyperemia. The NMES was performed using Revolution Wireless’s (Chattanooga, TN) “muscle pump” activation pattern. The “muscle pump” activating pattern continuously stimulates the muscle at 5 Hz with a biphasic pulse (duration/interval = 200/100 μsec). The current intensity was adjusted for each individual to produce muscle twitches at the maximal tolerable intensity. For the measurement of NIRS OXPHOS, the participants performed 20-30 seconds of NMES, which was immediately followed by a series of 20 arterial occlusions (~300 mmHg) to measure the rate of recovery of mVO_2_ as described previously ^1, 2^. The first 9 cycles were 5 sec “on,” 5 sec “off,” and the last 11 cycles were 10 sec “on,” 10 sec “off” (Note: one participant performed ~7 seconds of maximal isometric knee extension instead of the NMES). The NIRS-OXPHOS protocol was repeated three times with at least 5 minutes between trials. The NIRS-OXPHOS protocol was conducted on both legs.

The *mVO_2_* recovery kinetics were fit using custom-built MATLAB (The Mathworks, Natick, MA) routines written by Terence Ryan ^1, 3^. The *mVO_2_* for each recovery occlusion was measured after applying a blood volume correction and an ischemic calibration as described previously ^1, 3^. Specifically, we measured the negative slopes for the O_2_Hb for ~2.5-3 sec for the first nine 5 sec recovery occlusions and ~5-6 sec for the last three 10 s recovery occlusions. The *mVO_2_* slopes were subsequently fit to the following mono-exponential function: y = End – (Δ x e^-kt^) within the custom-built MATLAB program described extensively by Ryan and colleagues ^1, 3^. In brief, the “y” represents (blood volume corrected) *mVO_2,_* Δ represents the change in *mVO_2_* from rest to the end muscle activation (e.g., NMES), and k represents the rate constant, while “t” is the time ^1, 3^. The primary outcome from the present analysis was the Rate Constant from the mVO2 recovery kinetics. The rate constant equals 1/t*60 or 1/min, which serves as a measure of mitochondrial OXPHOS capacity. The rate constants included in the present analysis are the average of duplicate/triplicate measures. The rate constants were excluded from analysis if the R^2^ of the monoexponential fit was < 0.9, the coefficient of variation exceeded 20%, or the mVO_2_ recovery kinetics failed to fit a monoexponential curve. After excluding low-quality fits, the average R^2^ was ~0.98, and the coefficient of variation was ~9%. As a result, 8 participants per treatment group had complete pre- and post-intervention NIRS OXPHOS data.

References:

1. Ryan TE, Erickson ML, Brizendine JT, Young HJ, McCully KK. Noninvasive evaluation of skeletal muscle mitochondrial capacity with near-infrared spectroscopy: correcting for blood volume changes. J Appl Physiol (1985). 2012;113(2):175-83. PMC3404707.

2. Hanna R, Gosalia J, Demalis A, Hobson Z, McCully KK, Irving BA, Mookerjee S, Vairo GL, Proctor DN. Bilateral NIRS measurements of muscle mitochondrial capacity: Feasibility and repeatability. Physiol Rep. 2021;9(8):e14826. PMC8095363.

3. Ryan TE, Brizendine JT, McCully KK. A comparison of exercise type and intensity on the noninvasive assessment of skeletal muscle mitochondrial function using near-infrared spectroscopy. J Appl Physiol (1985). 2013;114(2):230-7.
